# Case series: Portomesenteric venous thrombosis complicating laparoscopic bariatric procedures

**DOI:** 10.1016/j.ijscr.2019.08.019

**Published:** 2019-08-20

**Authors:** Marwan Mohamed Bucheeri, Abdulmenem Yahya Abulsel

**Affiliations:** King Hamad University Hospital, House 2415, Road 4571, Block 745, Sanad, Bahrain

**Keywords:** Bariatric surgery, Obesity, Sleeve gastrectomy, Roux en y, Gastric, Bypass, Minigastric, Complications, Mesenteric, Portal, Portomesenteric, Splenic, Vein, Thrombosis

## Abstract

**Introduction:**

Portomesenteric vein thrombosis is a rare but documented complication of laparoscopic surgery. This rare complication is currently being encountered more frequently with the increase in the rates of laparoscopic bariatric surgery procedures being performed worldwide.

**Methods:**

A retrospective analysis was performed on all bariatric procedures performed in our center between July 2012 & December 2017 to identify cases complicated by portomesenteric venous thrombosis. The cases were compared in terms of operative details, patient presentation, diagnosis, patient risk factors for developing thrombosis, demographics and thrombophilia analysis & subsequent treatment and prognosis.

**Results:**

A total of 1030 bariatric procedures were performed between July 2012 & December 2017. Portomesenteric venous thrombosis complicated 3 of these cases (0.29%). Two of these cases had underwent a laparoscopic sleeve gastrectomy while the third had underwent a gastric band removal and a conversion to a single anastomosis gastric bypass. Amongst these patients, 2 were female while 1 was male with an average BMI 38.9 kg/m^2^. Only one of these patients was a smoker while none of them tested positive for thrombophilias. The diagnosis of portomesenteric venous thrombosis was confirmed with a contrast CT of the abdomen as all patients were re-admitted between 4–20 days post operatively after being discharged on postoperative day 2. All three cases were managed with systemic anticoagulants and none underwent invasive procedures or were re-explored surgically. All were subsequently discharged in good condition.

**Conclusion:**

Portomesenteric venous thrombosis is an uncommon yet potentially fatal complication of bariatric surgery. A high index of suspicion, early diagnosis and subsequent adequate management is required. Based on this case series and the potential risk of portomesenteric venous thrombosis, we altered our clinical practice to include a 1 week course of low molecular weight heparin to be administered to all patients after discharge.

## Highlights

• Portomesenteric vein thrombosis is a rare but potentially fatal complication of bariatric surgery.
• Portomesenteric venous thrombosis complicated 3 of 1030 cases (0.29%).
• Management is primarily with anticoagulants, but surgery is required if the patient shows signs of sepsis.
• A high index of suspicion, early diagnosis and subsequent adequate management is required.

## 1 Introduction

Bariatric surgery has been undertaken with increasing frequency worldwide. In 2013, statistics show that a total of 468,609 reported cases were performed world-wide [1] compared to 344,221 in 2008 [2]. The overall complication rate following bariatric surgery is reported at 10–17% [3].

Portomesenteric vein thrombosis is a rare yet documented complication of laparoscopic surgery [4]. This condition has been described after bariatric surgery with incidence reported between 0.2% and 1% [[5], [6], [7], [8]]. The risk factors for developing venous thromboembolisms (VTE) include oral contraceptives, smoking, hypercoagulable states and recent surgical interventions [9]. Obesity in itself is considered an independent risk factor predisposing to deep venous thrombosis thus increasing the risks of portomesenteric venous thrombosis in patients undergoing bariatric operations [10].

Portomesenteric vein thrombosis (PMVT) is a serious complication and can lead to death [8]. We present 3 cases encountered in our experience of over 1000 bariatric cases. We believe that bariatric surgeons worldwide need to be well aware with the presentation, and management strategy associated with this rare but potentially life threatening complication.

## 2 Case reports

### 2.1 Case 1

Patient 1 is a 24 years old female who is not a known case of any medical illness. She was admitted for an elective laparoscopic sleeve gastrectomy with a BMI of 40.6 kg/m^2^ and a weight of 118.3 kg. The procedure was uneventful. Her post-operative course was uneventful and she was discharged home on the second post-operative day. No anticoagulants were prescribed upon discharge.

The patient was readmitted 17 days post-operatively complaining of upper abdominal pain worsening with diet and associated with nausea and vomiting. Her abdomen was soft and lax with minimal tenderness in the right upper quadrant. An abdominal ultrasound was performed that demonstrated portal vein thrombosis. The patient was treated with therapeutic doses of low molecular weight heparin. The patient improved symptomatically and enoxaparin was bridged with warfarin. The patient was subsequently discharged after achieving a therapeutic INR. The warfarin was continued for a total of 3 months. A repeat abdominal US repeated 6 months later and showed a fully patent portal vein.

### 2.2 Case 2

Patient 2 is a 38 years old female who is not a known case of any medical illness. She has a history of a laparoscopic band insertion in 2012. The band was removed and the patient underwent a laparoscopic sleeve gastrectomy in 2014. Both these operations were undertaken in other centers and she failed to lose weight. She was admitted for an elective conversion from a sleeve gastrectomy to a minigastric (single anastomosis) bypass with a BMI of 36.05 kg/m^2^ and a weight of 90 kg. Intraoperatively, dense adhesions were noted between the stomach, liver and spleen. Bleeding was encountered as these adhesions were dissected. This was controlled with clip application. The operation was completed laparoscopically. Post-operatively, the patient’s repeat haemoglobin level on the first post-operative day was 7.9 g/dL dropping from 10.9 g/dL preoperatively. The patient was transfused 2 units of packed red blood cells and was discharged on the third post-operative day. Anticoagulation was not withheld.

The patient was readmitted 11 days post-operatively with complaints of left upper quadrant pain radiating to the left shoulder and neck associated with a fever measured at 38.5 °C. A CT abdomen & pelvis with IV and oral contrast showed thrombosis of the extrahepatic portal vein confluence, superior mesenteric and splenic veins. It also demonstrated a left subdiaphragmatic collection as well as an enlarged and ballooned spleen that was likely infarcted & liquefied. A radiologically guided drain was inserted and the collection drained leading to the resolution of the patient’s symptoms. She was subsequently discharged in good condition after achieving a therapeutic INR level. The warfarin was continued for 3 months. A repeat abdominal US performed 3 months after diagnosis of the portomesenteric venous thrombosis showed a patent portal vein measuring 1.1 cm in diameter.

### 2.3 Case 3

Patient 3 is a 28 years old male who is a known case of diabetes mellitus. He was admitted for an elective laparoscopic sleeve gastrectomy with a BMI of 40.3 kg/m^2^ and a weight of 125.2 kg. The procedure was performed successfully with no intraoperative complications noted. The post-operative course was uneventful. The patient was subsequently discharged in good condition on the second post-operative day with no anticoagulation prescribed on discharge.

The patient was readmitted 9 days post-operatively. He presented to the emergency department complaining of recurrent colicky severe central abdominal pain radiating to both lumbar regions. This was associated with nausea but no vomiting, fever or changes in bowel habits. He was tolerating oral fluid diet well. Examination was unremarkable with a totally soft and lax abdomen with no area of guarding or tenderness. A CT scan of the abdomen & pelvis was performed with IV and oral contrast and showed complete thrombosis of the main portal vein, right and left portal veins, splenic vein and superior mesenteric vein. The patient was started on a therapeutic dose of enoxaparin that was bridged with warfarin. He was discharged in good condition after achieving a therapeutic INR. Follow up in the clinic shows that the patient’s symptoms have fully resolved and he is tolerating oral diet well with no abdominal pain, nausea or vomiting.

## 3 Case analysis

The research work has been reported in line with the PROCESS criteria [11]. A total of 1030 bariatric procedures have been performed in out center. These include 769 sleeve gastrectomies, 53 biliopancreatic diversions, 120 minigastric bypass, 37 sleeve with bipartition, 27 roux-en-Y gastric bypass and 24 re-operations. Out of these operations, 3 were complicated with portomesenteric venous thrombosis (0.29%). There were no mortalities associated with this complication. All three cases were treated conservatively with systemic anticoagulation and none re-operated.

When comparing patient demographics (Table 1), we find that two of the patients are female, while one is male. The average age amongst the 3 patients is 30 years ranging between 24–38 years. The average BMI is 38.9 kg/m^2^ (between 36.05–40.6 kg/m^2^) with an average weight of 111.2 kg (between 90–125 kg). Only one of the patients is a smoker and none used oral contraceptives pre-operatively. None of the patient had a personal or family history of deep vein thrombosis (Table 2, Table 3).

**Table 1:** Summarizes the demographics of the 3 patients complicated by porto-mesenteric venous thrombosis.

Case	Gender	Age	Weight (kg)	BMI (kg/m^2^)	Comorbidities	Smoking Status
1	Female	24	118.3	40.6	–	–
2	Female	48	90.0	36.0	–	–
3	Male	28	125.2	40.3	Diabetes Mellitus	+

**Table 2:** Summarizes the operative data for the 3 cases.

Case	Operation	Operative Time (min)	Number of Ports	Intraoperative Complication
1	Laparoscopic Sleeve Gastrectomy	42	3	–
2	Laparoscopic conversion from Sleeve Gastrectomy to Minigastric Bypass	102	4	Intraoperative Bleeding
3	Laparoscopic Sleeve Gastrectomy	37	3	–

**Table 3:** Summarizes the Secondary Admission for all 3 patients with Porto-mesenteric Vein Thrombosis.

	Case 1	Case 2	Case 3
Readmission (Post Operative Day)	17	11	9
Symptoms			
• Abdominal Pain • Fever • Nausea • Vomiting	• RUQ • + • + • –	• LUQ • + • – • –	• Bilateral Flanks • – • + • –
Hb (g/dL)	12.4	10.5	15.1
Hct (%)	36.0	32.4	45.7
WBC (×10^3^/mm^3^)	8.5	30.0	5.0
CRP (mg/L)	180	295	35
Uric Acid (umol/l)	520	467	227
Creatinine (umol/l)	33	56	85
ALT / AST (u/l)	46 / 27	21 / 23	132 / 50
ALP / GGT (u/l)	107 / 61	125 / 29	97 / 99
TBili / Dbili (umol/l)	11.1 / 4.1	9.6 / 3.8	23.4 / 8.9
Urinalysis			
• Leukocyte Esterase • Nitrites • Ketones	• Negative • Negative • >150	• Negative • Negative • >150	• Negative • Negative • 60
Diagnosis Modality	Abdominal US	CT Abdomen	CT Abdomen
Symptom Resolution (Days)	2	2	4
Duration of Admission (Days)	5	10	9

All patients received the same pre-, intra- and post-operative deep vein thromboprophylaxis protocol. Pre-operatively, gradual compression stockings were utilized from admission until discharge. All patients received 5000 iU of Heparin on the day of surgery at 6 AM. Intraoperatively, the patient was placed on pneumatic compression stockings. Post-operatively, heparin 5000 iU was started 8 h after the operation and administered every 8 h. This was switched to enoxaparin 40 mg on the first post-operative day. Patients are mobilized with assistance within 4–6 h from the operation. Pneumatic compression stockings were applied throughout the admission whenever the patient was in bed.

Among the 3 patients, intraoperative complications were only encountered with one patient. Two of the patients underwent a laparoscopic sleeve gastrectomy while one underwent a conversion from a sleeve gastrectomy to a minigastric bypass. The complication was encountered with the latter case due to dense adhesions between the stomach and the spleen face while trying to mobilize the stomach. Injury a short gastric artery led to the patient bleeding around 600 ml of blood. No intraoperative blood transfusion was required. The bleeding was controlled laparoscopically with the use of clips and the case was not converted into a laparotomy. The average operative time was 60 min ranging between 37–102 min.

Post operatively, the two patients who underwent a sleeve gastrectomy had an uneventful course. They were both discharged on the second post-operative day. The third patient had a drop in haemoglobin to 7.9 g/dL and was transfused 1 unit of blood. She was subsequently discharged on the third post-operative day with a haemoglobin level of 9.8 g/dL. All the patients were on intravenous fluids throughout admission and underwent a gastrograffin contrast study on post-operative day 1. None of the patients complained of severe abdominal pain during the initial admission and no complications were noted during the hospitalization period.

The readmission period was between 9 and 17 days postoperatively, with all patients complaining of abdominal pain on their presentation. The patient’s symptoms were out of proportion when compared to their clinical findings. All patients maintained a soft and lax abdomen with minimal tenderness elicited in the upper abdomen. The laboratory findings elicited a high CRP for all 3 patients while the WBC count was only elevated in one. The lab findings demonstrated dehydration in all patients with a high uric acid level and ketones in the urine.

Diagnosis was established with a CT scan in 2 of the 3 patients while an abdominal US established the diagnosis in the third. Upon diagnosis, all three patients were managed conservatively and started on therapeutic doses of enoxaparin that was bridged with warfarin therapy. Symptoms resolved in all patients between 2–4 days. One of the patient required a radiological guided drainage of a sub-diaphragmatic collection yielding a total of 400 ml of bloody output over 3 days. None of the patients were re-operated. Subsequently, all of the patients were discharged in good condition to outpatient follow up in an average of 8 days (range 5–10 days). All of the patients continued oral anticoagulation therapy for a total of 3 months. Subsequent thrombophilia analysis was negative in all patients for Protein C deficiency, Protein S deficiency & Factor V Leiden.

On subsequent follow up visits, the patients remained completely asymptomatic. An abdominal ultrasound was performed in two of the patients to assess the patency of the portal vein after the period of anticoagulation. Both ultrasounds showed fully patent portal veins for both patients. The third patient lost follow up and no repeat imaging was performed.

## 4 Discussion

Venous thrombosis is described as a multifactorial disease of multiple etiologies both systemic and focal [12]. In a study amongst patients with portal vein thrombosis, it was found that 41% of patients had one or more prothrombotic coagulation disorder with 8.3% of patients had a multimodal etiology for thrombosis that included a coagulation factor abnormality and a primary myeloproliferative disease. A regional precipitating factor was identified in 27.7% of these patients [13].

Multiple factors can lead to portomesenteric venous thrombosis following laparoscopic surgery. Patient factors include undiagnosed thrombophilias like Protein S & Protein C deficiency [13], polycythemia vera, thrombocytosis and pre-operative oral contraceptive use [8].

Amongst bariatric patients, obesity in itself is considered a pro-thrombotic state predisposing patients to DVT. Venous stasis has been attributed to a sedentary life style and increased intra-abdominal pressures in the obese [14]. Also, obese patients are described to be in a state of chronic systemic inflammation predisposing them to DVTs [15]. Multiple studies demonstrated the correlation between obesity and VTE with Ageno et al. demonstrating a significant increase by a factor greater than 2 in rates of spontaneous VTE between those with a normal BMI and the obese [16]. Some studies attributed the increase in rate of idiopathic VTE to central obesity [17,18]. Obesity has also been attributed to recurrent VTE with Eichinger et al. demonstrating the probability of VTE recurrence at 9.3% (95% CI, 6.0%–12.7%) in patients with a normal BMI, 16.7% (95% CI, 11.0%–22.3%) in the overweight and 17.5% (95% CI, 13.0%–22.0%) amongst the obese [19].

Intraoperative factors can also play a role in PMVT. Patients with morbid obesity have an intra-abdominal pressure 2–3 times higher than that of non-obese patients [20]. Upon insufflation of the peritoneum, it was found that changes occur in the splanchnic blood flow. The rise in intraperitoneal pressure leads to a progressive decrease in portal vein blood flow contributing to venous stasis hence theoretically predisposing to PMVT [20,21]. The clinical significance of this phenomenon is controversial in literature [22]. Venous stasis has also been linked to the patient’s intraoperative position. A prolonged duration in the reverse Trendelenberg position being attributed to deep venous thrombosis [4].

Intraoperative manipulation of the splanchnic vasculature may play a crucial role in PMVT [23]. For example, PMVT has been well described post splenectomy [24] as ligation of the splenic vein predisposes to endothelial damage predisposing to venous thrombosis within the vein [23]. Ikeda et al. went further and demonstrated a higher incidence of portal vein thrombosis following laparoscopic rather than open splenectomies [40]. During bariatric surgery, and when manipulating the stomach, thermal or mechanical injury to the splenic or mesenteric veins can induce venous thrombosis causing the propagation of the thrombus proximally leading to PMVT [7].

Post operatively, dehydration is a major factor that can lead to DVT. Due to the nature of bariatric procedures, oral fluid intake is markedly reduced in the first few weeks post operatively. This is evident in all 3 cases presented as all three demonstrated laboratory findings indicating dehydration. This condition leads to hyper-viscosity predisposing the patient to the risk of PMVT. Dehydration has been described by Goitein et al. and Rottenstreich et al. as a contributing factor to PMVT [25,26].

During our literature search, we encountered many case series and case reports documenting PMVT as a complication following laparoscopic bariatric surgery [[4], [5], [6], [7], [8],^23^,[25], [26], [27], [28], [29], [30], [31], [32], [33], [34], [35], [36], [37], [38], [39]].

PMVT, although a rare complication, it has been described primarily in patients undergoing a sleeve gastrectomy. It has also been described in patients following roux-en-Y gastric bypass [4,8,28,29,32], adjustable gastric banding [25] & biliopancreatic diversion [36]. Carlin et al. studied over 52,000 patients following bariatric procedures. He concluded that the rates of PMVT are lower than those of deep vein thrombosis and pulmonary embolisms standing at 0.04% compared to 0.21% & 0.18% respectively. The analysis also demonstrated the rise in the rates of PMVT over a period of 10 years from 2006–2015. He concluded that a sleeve gastrectomy stands alone as an independent risk factor for developing PMVT [33].

BMI does not appear to be of significance when considering patients at risk of developing PMVT. Cesaretti et al. reported a PMVT in patients with a BMI of 28.4 and 35.4 kg/m^2^ [36]. Villagrán et al. reported 5 cases of PMVT following sleeve gastrectomies and the mean BMI was calculated at 38.5 kg/m^2^ (36.1–40.9 kg/m^2^) [7]. On the other hand, Swartz et al. reported 3 cases of PMVT following roux-en-Y gastric bypasses with an average BMI of 56.6 kg/m^2^ [8] while Sonpal et al. reported a case with a BMI of 63 kg/m^2^ [32]. This is more evident in the case series of Bellanger et al. where he reported 3 cases of PMVT in patients with a BMI of 36, 38 & 53 kg/m^2^ [30]. Hence, these series of cases do not demonstrate the BMI as a primary etiological or predictive factor for the development of PMVT.

Patients who develop PMVT have a wide variety of presentations. Most patients develop non-specific generalized abdominal pain and this was evident in our cases [41]. Patients can also develop nausea, vomiting & GI bleeding with the physical examination ranging from totally unremarkable to peritonitis and septic shock [25,42]. The presentation period also varies significantly with Salinas et al. presenting 17 patients with PMVT following bariatric surgery and demonstrating a median presentation period of 15 days post operatively with a range from 8 to as long as 43 days post operatively [6].

Upon presentation, a contrast enhanced CT is the radiological investigation of choice in establishing a diagnosis of PMVT [42]. Two of our patients had underwent this modality upon presentation while the third underwent an abdominal doppler US that revealed PMVT. Although the doppler ultrasound is specific, it is not as sensitive as a CT scan of the abdomen [41]. During the acute presentation, laboratory findings do not correlate with a diagnosis of PMVT although leukocytosis and hemoconcentration are considered common findings [43].

After establishing the diagnosis of PMVT, blood tests are usually acquired to test for hypercoagulable states like Protein C or S deficiency, prothrombin gene mutation, factor V leiden and antithrombin III deficiency [43]. Anticoagulation is initiated immediately either as IV unfractionated heparin or low molecular weight heparin [44,45]. A study by Condat et al. showed that portal & superior mesenteric vein recanalization rates are over 85% in patients initiated on anticoagulation following PMVT. The author went further to state that the probability of recanalization was related to the extent of the thrombosis [46]. Oral anticoagulation is usually prescribed for 6 months to prevent recurrence and sometimes continued lifelong if a hypercoagulable state is identified [43].

Patients with signs of peritonitis require intervention. Swartz et al. described 3 cases of PMVT requiring surgical intervention following roux-en-Y gastic bypass. One patient underwent small bowel resection after necrotic bowel was found intraoperatively, while another underwent a diagnostic laparoscopy only to assess for the presence of necrosis. The third patient had 2 exploratory laparotomies and extensive stomach, bowel and colonic necrosis was identified and the patient succumbed to sepsis [8]. Goiten et al., Darcy et al., Johnson et al., Keung et al., Muneer et al., Roy et al. and Cesaretti et al. all reported cases requiring surgical intervention after diagnosing PMVT with the commonest intraoperative finding being bowel necrosis [25,27,29,[34], [35], [36],^39^]. Bowel resection & primary anastomosis is the surgical option of choice in such cases [43].

Selective thrombolysis had also been described as a modality of treatment for PMVT. This can be achieved by 2 methods, the first of which is operatively placed catheters. This has been described by Kaplan et al. where they achieved recanalization of the right portal and superior mesenteric veins by infusing rTPA into a jejunal vein [47]. The second is through interventional radiology where thrombolytic therapy was infused into the portal system via percutaneous transhepatic catheters or through mesenteric venous access gained via the femoral or jugular veins [[48], [49], [50], [51]]. This treatment remains controversial. Smalberg et al. reported a series of 12 patients with splanchnic venous thrombosis managed by selective thrombolysis. The thrombolysis was successful in 3 patients, partially successful in 4 patients with 2 reported mortalities due to procedure-related bleeding [52].

PMVT related mortalities have been described in patients following bariatric surgery [5,8,34,35]. Reports of PMVT show that the mortality rates are around 10–20% [43]. Amitrano et al. showed that 87.5% of deaths related to splanchnic vein thromboses was cased in the acute presentation as a result of intestinal infarction [53]. Recurrence of PMVT has also been described and reported at 6.5% and oral anticoagulation was found to play an important role in preventing recurrence [54].

## 5 Conclusion

Portomesenteric venous thrombosis is a rare but serious complication that is well documented following bariatric surgery. With the rise in rates of bariatric procedures worldwide, especially sleeve gastrectomies, surgeons need to be aware of this potentially fatal complication. Several risk factors prove to be contributory to the development of PMVT ranging from thrombophilia to intraoperative splanchnic vasculature manipulation, to postoperative dehydration. A high index of suspicion & a prompt diagnosis with a CT scan of the abdomen is essential for subsequent management. In our series, treatment involved anticoagulation with no surgical intervention warranted. However, treatment should be individualized and may include surgical exploration. Based on this case series we have altered our clinical practice to include a 1 week course of low molecular heparin to be prescribed upon discharge of all our bariatric patients.

## Funding

None.

## Ethical approval

The case report was approved by the Ethics Committee in King Hamad University Hospital in the Kingdom of Bahrain.

## Consent

Written informed consent was obtained from the patients for publication of this case report and accompanying images. A copy of the written consent is available for review by the Editor-in-Chief of this journal on request.

## Author contribution

Dr. Marwan Mohamed Bucheeri & Dr. Abdulmenem Abulsel.

Both authors were fully involved in data collection and reporting of the cases.

Both authors were fully involved in researching the literature for such complications and were fully involved in writing the discussion and conclusion of the case series.

## Registration of research studies

researchregistry4939.

## Guarantor

Dr. Marwan Mohamed Bucheeri.

## Provenance and peer review

Not commissioned, externally peer-reviewed.

## Declaration of Competing Interest

None.
